# Modeling the Electrophysiological Properties of the Infarct Border Zone

**DOI:** 10.3389/fphys.2018.00356

**Published:** 2018-04-09

**Authors:** Caroline Mendonca Costa, Gernot Plank, Christopher A. Rinaldi, Steven A. Niederer, Martin J. Bishop

**Affiliations:** ^1^School of Biomedical Engineering and Imaging Sciences, King's College London, London, United Kingdom; ^2^Department of Biophysics, Medical University of Graz, Graz, Austria; ^3^Cardiac Department, St. Thomas' Hospital, London, United Kingdom

**Keywords:** cardiac electrophysiology, myocardial infarct, infarct border zone, gray zone, computational modeling

## Abstract

Ventricular arrhythmias (VA) in patients with myocardial infarction (MI) are thought to be associated with structural and electrophysiological remodeling within the infarct border zone (BZ). Personalized computational models have been used to investigate the potential role of the infarct BZ in arrhythmogenesis, which still remains incompletely understood. Most recent models have relied on experimental data to assign BZ properties. However, experimental measurements vary significantly resulting in different computational representations of this region. Here, we review experimental data available in the literature to determine the most prominent properties of the infarct BZ. Computational models are then used to investigate the effect of different representations of the BZ on activation and repolarization properties, which may be associated with VA. Experimental data obtained from several animal species and patients with infarct show that BZ properties vary significantly depending on disease's stage, with the early disease stage dominated by ionic remodeling and the chronic stage by structural remodeling. In addition, our simulations show that ionic remodeling in the BZ leads to large repolarization gradients in the vicinity of the scar, which may have a significant impact on arrhythmia simulations, while structural remodeling plays a secondary role. We conclude that it is imperative to faithfully represent the properties of regions of infarction within computational models specific to the disease stage under investigation in order to conduct *in silico* mechanistic investigations.

## 1. Introduction

Cardiovascular disease represents 31% of all worldwide mortality with Coronary Heart Disease (CHD) responsible for an estimated 41% of these deaths (WHO, 2015). CHD results from the obstruction of a coronary artery causing ischemia, i.e., the interruption of blood supply to the myocardium, which can cause severe ventricular arrhythmias (VA) and sudden cardiac death (SCD) (Janse and Wit, 1989; Rodríguez et al., 2006; Ferrero et al., 2014) as well as irreversible tissue damage, known as myocardial infarction (MI).

Arrhythmias associated with CHD are generally thought to be due to re-entry, however, the cause and specific type of re-entry varies over time after ischemia onset. Specifically, CHD can be divided into three phases according to the type of VA most commonly observed, namely, the acute, sub-acute and healing/healed phases. The acute phase comprises the first hour after ischemia onset. VA in this phase is due to functional re-entry (Liang et al., 2013) or spontaneous activity (Pollard et al., 2002). The sub-acute phase comprises the next 72 h after ischemia onset and VA in this phase is thought to be due to abnormal automaticity of Purkinje cells that survive in the subendocardium (Fenoglio et al., 1976; Liang et al., 2013). In patients who survive acute and sub-acute ischemia, the myocardium begins to heal. During the healing phase, dead cardiomyocytes are slowly replaced by collagen leading to the formation of a scar with a dense collagenous core surrounded by a thin layer of surviving myocardium, known as the infarct border zone (BZ). Several studies have reported altered electro-anatomical properties at the BZ of healing and healed (chronic) infarcts (Gardner et al., 1985; Ursell et al., 1985), which may be associated with the development of re-entry (Kléber and Rudy, 2004). However, the underlying mechanisms are not fully understood and may differ depending on the specific electro-anatomical changes that occur within the BZ.

The risk of VA is high among patients with chronic infarct and about 12.5% suffer from SCD 2 years post-MI (Raviele et al., 1998). Thus, risk stratification is crucial to identify patients at high risk of developing VA to plan appropriate therapy. Currently, identification of high risk patients largely relies on global measures of left ventricular (LV) function, which poorly reflect electro-anatomical changes underlying the formation of arrhythmias in MI patients. Scar tissue heterogeneity, measured from late gadolinium enhancement (LGE) magnetic resonance imaging (MRI) as the volume of BZ relative to LV volume, has recently been shown to predict VA risk in patients with chronic MI (Schmidt et al., 2007; Kwon et al., 2015) and may improve risk stratification. More recently, computer models built upon patient specific anatomy have been used to predict VA risk in patients with chronic MI with very promising results. Specifically, the Virtual-heart Arrhythmia Risk Predictor (VARP) was shown to be a better predictor of VA than scar heterogeneity and other conventional indices in a cohort of 41 patients Arevalo et al. (2016). Similar approaches have also been employed by other groups to study the effect of scar morphology on VA inducibility (Ringenberg et al., 2012) and to predict VA inducibility and circuit morphology (Chen et al., 2015).

Computer models are a promising strategy to study arrhythmia mechanisms as well as predict arrhythmia risk, as described above. However, building personalized ventricular computer models of patients with MI remains a challenging task. It requires identifying (segmenting) the anatomy of the heart, the infarct scar and the BZ from imaging data and combining it with a mathematical description of the patient's EP based ideally on non-invasive, clinical data, such as the electrocardiogram (ECG). However, the ECG does not allow identifying specific regional differences in EP properties, such as action potential duration (APD) and conduction velocity (CV). Particularly, in the case of computer models of MI, identifying the specific EP properties of the BZ from the ECG is virtually impossible, as the BZ can be as thin as a few hundred micrometers Bakker et al. (1988). Consequently, most computer models rely on experimental data to assign BZ properties. However, experimental results are afflicted with significant uncertainty and may vary depending on the animal species and experimental conditions. As a result, recent image-based computer models of MI have chosen different computational representations of the EP properties of the BZ (McDowell et al., 2011; Ringenberg et al., 2012; Sermesant et al., 2012; Arevalo et al., 2013). These may largely affect generation and sustenance of VA (Cabo and Boyden, 2003; Decker and Rudy, 2010), thus, having important consequences in the interpretation of VA simulations.

The aim of this article is two-fold. First, we review experimental data available in the literature to identify which EP properties of the BZ are most consistently reported and, thus, would be most appropriate to include in computer models of MI. Finally, we use idealized 2D models of scar and BZ to quantify the consequences of different computational representations of the BZ in repolarization characteristics, which may play a role in VA simulations.

## 2. Review of experimental data of the electrophysiological remodeling within the infarct border zone

In this section, we investigate the EP properties of the infarct BZ based on experimental data on action potentials and ionic currents characteristics as well as morphological properties available in the literature. We focus on data obtained during and after scar formation, which occurs over the first few days or the first weeks after infarction depending on the species (Richardson et al., 2015). Reviews on simulation and experimental studies during acute ischemia can be found elsewhere (Rodríguez et al., 2006; Ferrero et al., 2014).

**Figure 1:**
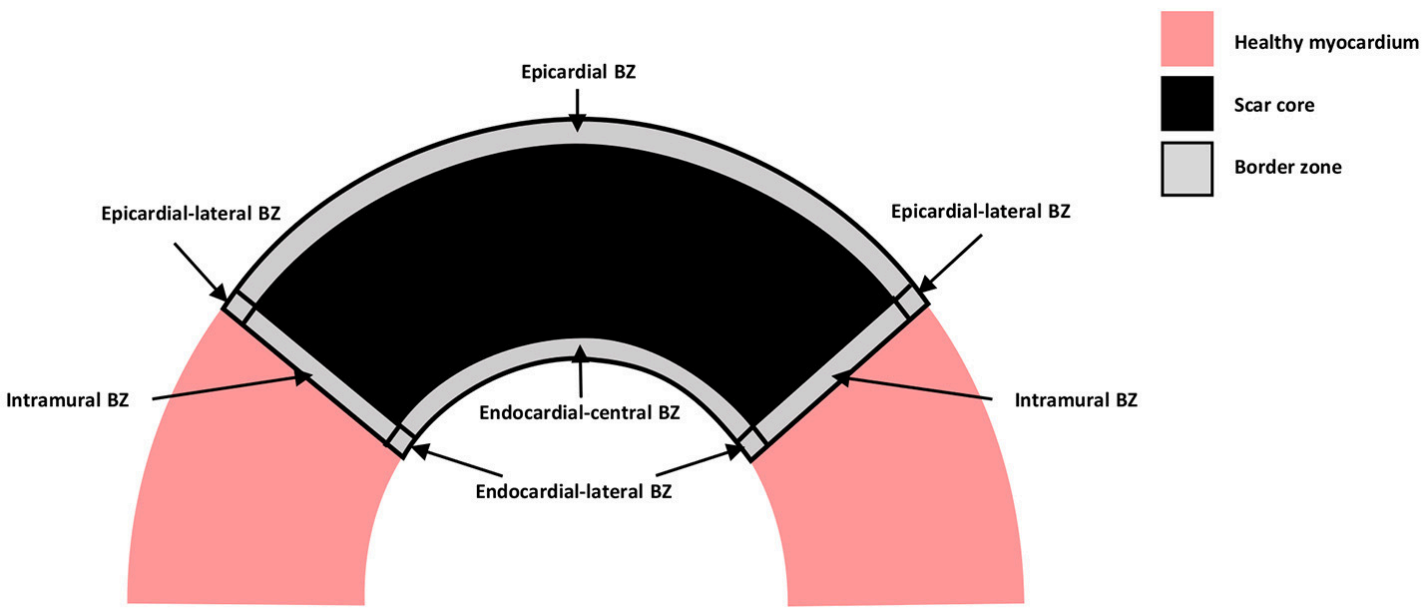
Schematic representation of the distinct types of BZ. Healthy myocardium is represented in pink, scar core in black and BZ in gray.

A schematic representation of the distinct types of BZ are shown in Figure 1, where myocardium is represented in pink, the scar core in black and the BZ in gray. The epicardial BZ refers to the layer of myocardium that survives below the epicardial surface, the endocardial BZ refers to the layer that survives below the endocardial surface, and the intramural BZ refers to the BZ in the mid-myocardium surrounding the scar core. As shown in Figure 1, the endocardial BZ is divided into the endocardial-central BZ and the endocardial-lateral BZ. Although the epicardial BZ may also contain a lateral BZ, this region was not identified in our literature review. However, we illustrate an epicardial-lateral BZ here for the sake of completeness. Similarly, an intramural BZ is also illustrated in Figure 1, although no measurements in this region were found in the literature. When the BZ region was not specified in a study, we use the broad term BZ. While some of the studies included in our review may have confounded BZ with scar isthmus measurements, this was not clear from their methods and so measurements were assumed to be representative of a homogeneous BZ region. As such, we illustrate the scar and BZ as homogeneous structures, without any islands of surviving tissue or isthmuses.

Qualitative findings from the experimental studies found in the literature are summarized in Table 1. The species, the BZ region from where samples were obtained, the time period when recordings were made, the EP properties, and the structural properties found in each study are shown. Results range from data obtained *in vivo* and *ex vivo* from canine, feline, swine, murine, and rabbit models of infarct, as well as *ex vivo* data from patients with prior MI. In the animal models, infarct was induced either via permanent ligation or temporary occlusion of one or more coronary arteries. EP recordings were obtained via several methods, such as micro electrodes, balloon electrodes, suction pipettes, and optical mapping. Morphology of the scars was analyzed via histological slices or confocal microscopy.

**Table 1:** Experimental data on EP and structural properties of the BZ.

**Study**	**Species**	**Stage**	**Region**	**EP prop**.	**Structural prop**.
Lue and Boyden, 1992	Dog	5 d	Epi BZ	↓I_*to*_ ↑APD_100_	–
				↓APA	
Pu and Boyden, 1997	Dog	5 d	Epi BZ	↓I_*Na*_	–
Jiang et al., 2000	Dog	5 d	Epi BZ	↓I_*Kr*_ ↓I_*Ks*_	–
Dun et al., 2004	Dog	5–14 d	Epi BZ	↓I_*to*_ ↓I_*CaL*_	–
		8 w		↓I_*CaL*_	
Baba et al., 2005	Dog	5 d	Epi BZ	↓I_*CaL*_ ↓I_*Na*_	–
				↓APD_90_	
Cabo et al., 2006	Dog	4–5 d	Epi BZ	–	↓G_*Trans*_
					GJ lat ↓CV
Ursell et al., 1985	Dog	5 d	Epi BZ	↓MUV ↓APD_90_	nCV
				↓ APA nEG	
		2 w		↓APD_90_	Fibrosis
		8 w		nAPD nMUV	↓CV iso
				nAPA	Disarray
					Fibrosis
Gardner et al., 1985	Dog	5 d	Epi BZ	↓MUV ↓APD_90_	nCV
				↓APA nEG	
		2 w		↓APD_90_ fEG	Fibrosis
		8 w		nAPD nMUV	↓CV iso
				nAPA fEG	Disarray
					Fibrosis
Spear et al., 1983	Dog	3–5 d	Epi BZ	↓MUV ↓APD_30_	–
				↓APA nAPD_100_	
		8–15 d		↓APD_30_ nAPD_100_	
Luke and Saffitz, 1991	Dog	3–10 w	Epi BZ	–	↓GJ density
					Fibrosis
Peters et al., 1997	Dog	4 d	Epi BZ	–	GJ lat
Yao et al., 2003	Dog	5 d	Epi BZ	–	↓G_*Trans*_
Pinto et al., 1997	Cat	2–4 m	EndoL BZ	↓I_*CaL*_	–
Kimura et al., 1986	Cat	2–6 m	EndoL BZ	↓APD_90_ ↓APA	–
			EndoC BZ	↑APD_90_	–
Wong et al., 1982	Cat	2–7 m	EndoL BZ	↓APD_90_	–
			EndoC BZ	↑APD_90_	–
Myerburg et al., 1982	Cat	2–4 m	EndoC BZ	↑APD_90_	Fibrosis
Kimura et al., 1988	Cat	2–4 m	EndoC BZ	nAPD_50_ nAPD_90_	–
Pop et al., 2012	Pig	4 w	Epi BZ	↓APD ↓MUV	↓CV Disarray
				↓APA	
Denisko et al., 2017	Pig	5 w	BZ	nAPD	–
Mills et al., 2005	Mouse	7 d	Epi BZ	nAPD_90_	↓CV_*Trans*_
					Fibrosis
Rutherford, 2013	Mouse	14 d	BZ	–	↓CV_*Trans*_
					Fibrosis
					Disarray
Weigand et al., 2016	Mouse	6 w	Epi BZ	nAPD_90_	–
Chou et al., 2007	Rabbit	7 d	BZ	↓APD_80_	–
Lee et al., 2013	Rabbit	5 w	BZ	↑I_*KAS*_ ↓APD_80_	–
Litwin and Bridge, 1971	Rabbit	8 w	BZ	↓I_*CaL*_ ↑APD_90_	–
Walker et al., 2007	Rabbit	8 w	BZ	Abnormal AP	↓CV
Spear et al., 1979	Human	1 m+	EndoC BZ	↑APD_90_	Fibrosis
Dangman et al., 1982	Human	12 m+	EndoL BZ	nAPD_90_	–
			EndoC BZ	↑APD_90_	–
Smith et al., 1991	Human	–	EndoC BZ	–	GJ lat Fibrosis
					Disarray
Kostin et al., 2003	Human	–	BZ	–	GJ lat Fibrosis
					Disarray
Bakker et al., 1988	Human	16 d+	EndoC BZ	fEG	↓CV_*Trans*_
					Disarray
					Fibrosis
Pogwizd et al., 1992	Human	4 m+	BZ	fEG	↓CV_*Trans*_
					Disarray
					Fibrosis

*Disease stage is presented in days (d), weeks (w), or months (m). In most studies, a distinction between epicardium (Epi) BZ, endocardial-central (EndoC) BZ, and endocardial-lateral (EndoL) BZ was made. EP properties (prop.) include action potential duration (APD), maximum upstroke velocity (MUV), action potential amplitude (APA), fast sodium current (I_Na_), L-type calcium current (I_CaL_), rapid and slow rectifying potassium currents (I_Kr_ and I_Ks_, respectively), the transient potassium current (I_to_), the Apamin-sensitive potassium current (I_KAS_) and electrograms (EG), normal (nEG) or fractionated (fEG). Structural properties include, gap junction (GJ) transverse conductance (G_trans_) and lateralization (lat), conduction velocity (CV), and the presence of fibrosis or (fiber) disarray. Arrows indicate increased (↑) or decreased (↓) quantities relative to normal myocardium, whereas “n” indicates normal or unchanged characteristics*.

In the next two sections we analyse the experimental data presented in Table 1. We divided our findings into infarct healing (less than 5 weeks after MI) and healed infarct (at least 5 weeks after MI), based on histological evidence from patients post-MI (Fishbein et al., 1978).

### 2.1. The infarct border zone during infarct healing

#### 2.1.1. Electrophysiological remodeling

The fast sodium current (I_*Na*_) is the main inward current during membrane depolarization and the main determinant of maximum upstroke velocity (MUV) and action potential amplitude (APA) in a single cell. Thus, a reduction in I_*Na*_ (Pu and Boyden, 1997; Baba et al., 2005) during infarct healing is directly associated with a slower MUV (Spear et al., 1983; Ursell et al., 1985) and reduced APA (Spear et al., 1983; Gardner et al., 1985; Lue and Boyden, 1992) at this stage of MI. A reduction in this current has also been associated with post-repolarization refractoriness (Lue and Boyden, 1992; Pu and Boyden, 1997; Baba et al., 2005).

Following the AP upstroke, I_*Na*_ starts to deactivate. In most mammals, with exception of the guinea pig (Varro et al., 1993), I_*Na*_ deactivation is accompanied by activation of the transient outward potassium current (*I*_*to*_). The latter is a repolarizing current and is responsible for the early repolarization phase (notch), which follows depolarization. Accordingly, a reduction in *I*_*to*_ (Lue and Boyden, 1992; Dun et al., 2004) is thought to be responsible for the absence of a notch in the AP of cells from the canine epicardial BZ (Lue and Boyden, 1992).

Depolarizing calcium currents are activated following early repolarization. These currents, particularly the L-type calcium current (I_*CaL*_), oppose repolarization and it is the balance between these and the repolarizing (potassium) currents that keeps the AP at a plateau in most species, except rat and mice (Varro et al., 1993). Thus, reduced density of the I_*CaL*_ will generally accelerate repolarization, shortening the APD. Accordingly, reduced density of I_*CaL*_ was observed in canine BZ (Dun et al., 2004; Baba et al., 2005) and was associated with APD shortening in this species (Baba et al., 2005). This current also plays a major role in calcium dynamics and its impairment is associated with dysfunctional myocyte contraction in rabbits (Litwin and Bridge, 1971).

When I_*CaL*_ deactivates, membrane repolarization begins. The rapid (I_*Kr*_) and slow (*I*_*Ks*_) components of delayed rectifier currents are the main repolarization currents, thus, reduced density of these currents (Jiang et al., 2000) slows repolarization causing APD prolongation. However, APD prolongation was reported only by one study during the healing phase (Lue and Boyden, 1992), while most studies report APD shortening (Spear et al., 1983; Gardner et al., 1985; Ursell et al., 1985; Baba et al., 2005; Chou et al., 2007; Pop et al., 2012) or even normal APD (Mills et al., 2005).

In general, changes in individual ionic currents should be analyzed with caution when studying changes in AP characteristics, such as APA, MUV, and APD, since it is the interaction between several currents which will determine AP characteristics. It is also important to realize that the data listed in Table 1 was obtained from a variety of species in which ionic currents are expressed to different extents leading to variations in overall AP characteristics (Varro et al., 1993). Thus, changes in a particular ionic current may have different relative effects between cells from different species. Nonetheless, the experimental data in Table 1 suggests that APD is generally shorter in the BZ during infarct healing despite individual changes in ionic currents and species differences.

#### 2.1.2. Structural remodeling

CV is influenced by several factors, such as I_*Na*_ density, GJ conductance and extracellular conductivity. Reduced I_*Na*_ density (Pu and Boyden, 1997; Baba et al., 2005) and decreased transverse GJ conductance (Yao et al., 2003) are associated with reduced CV in tissue when considered individually (Rohr et al., 1998; Dhillon et al., 2013). Conduction slowing and decreased GJ conductance were observed in canine epicardial BZ tissue 4–5 days after MI (Cabo et al., 2006). However, the relationship between the two was not clear, as GJ remodeling varied between different BZ regions and confounding factors such as ion channel remodeling may have affected measurements of CV. Conversely, no conduction slowing was observed in two other studies on canine epicardial BZ 5 days after MI (Gardner et al., 1985; Ursell et al., 1985). This may be explained by the fact that cells at the canine epicardial BZ were often separated by interstitial edema (Ursell et al., 1985), which will potentially increase extracellular conductivity and compensate for the conduction slowing caused by reduced I_*Na*_ density and GJ conductance (Cabo and Boyden, 2009). On the other hand, conduction slowing in the direction transverse to fibers was observed in murine at 7 (Mills et al., 2005) and 14 (Rutherford, 2013) days after MI, respectively. However, conduction slowing in these cases was associated with the presence of interstitial fibrosis, which interrupts propagation transverse to fibers causing local propagation delays. Interstitial fibrosis appears after 2 weeks in canine (Gardner et al., 1985; Ursell et al., 1985) and it was occasionally associated with electrogram fractionation, but no apparent conduction slowing at this stage (Gardner et al., 1985).

Spatial distribution of GJ also plays a role in CV. In normal tissue, GJ are mainly located at the ends of cells, called intercalated disks. This characteristic distribution is largely responsible for CV anisotropy, which is slower transverse to fibers and normal to the sheet orientation. Thus, it is intuitive that an increased concentration of GJ at the lateral boundaries of cells, known as GJ lateralization, would lead to decreased anisotropy of CV. GJ lateralization has been observed at the canine epicardial BZ 4 and 5 days after MI (Peters et al., 1997; Cabo et al., 2006) and was associated with improved conduction transverse to fibers (Cabo et al., 2006). However, it is also possible that some of the GJ located at the lateral of remodeled cells are not functional (Matsushita et al., 1999). Thus, fully understanding the role that GJ lateralization at the BZ may play on CV requires further studies.

### 2.2. The infarct border zone in healed infarcts

#### 2.2.1. Electrophysiological remodeling

##### 2.2.1.1. Epicardial and endocardial-lateral border zones

Calcium sensitive potassium currents are activated by calcium overload and blocked by Apamin (Adelman et al., 2012). Although these currents are not typically present in normal ventricles, the Apamin-sensitive potassium current (I_*KAS*_) has recently been found to be upregulated at the BZ of rabbits with healed infarcts (Lee et al., 2013). In addition, reduced I_*CaL*_ density has been reported in canine (Dun et al., 2004), feline (Pinto et al., 1997), and rabbits (Litwin and Bridge, 1971) with healed infarcts. Both upregulation of I_*KAS*_ and downregulation of I_*CaL*_ accelerate repolarization causing APD shortening. In fact, APD shortening was reported both in feline (Pinto et al., 1997) and rabbit (Lee et al., 2013) in association with these currents. APD shortening was also found in feline (Wong et al., 1982; Kimura et al., 1986), although measurements of neither I_*CaL*_ nor I_*KAS*_ were reported in those studies. On the other hand, the canine (Gardner et al., 1985; Ursell et al., 1985), porcine (Denisko et al., 2017), murine (Weigand et al., 2016), and human (Dangman et al., 1982) BZ of healed infarcts exhibit normal APD, while APD prolongation has also been found in rabbits with healed infarcts (Litwin and Bridge, 1971).

While altered I_*KAS*_ and I_*CaL*_ were associated with APD shortening in feline (Pinto et al., 1997) and rabbit (Lee et al., 2013), changes in individual currents only tell part of the story, as experimental conditions and the stage of MI may also influence APD. The latter is well exemplified in two studies (Gardner et al., 1985; Ursell et al., 1985), where canine epicardial BZ tissue was studied from 1 day to 8 weeks after MI. These studies report shorter APD at 1 day, 5 days and 2 weeks after MI, where APD is progressively shortened after MI and is shortest at 2 weeks. However, after 2 weeks, the APD begins to increase and returns to normal values after 8 weeks. Similarly, normal APD values were also found in murine epicardial BZ tissue 6 weeks after MI (Weigand et al., 2016), suggesting that the healing process is complete earlier in this species.

Rabbits are a particular case among the studies shown in Table 1, as they exhibit a shorter APD_80_ 5 weeks after MI (Lee et al., 2013), but a longer APD_90_ by 8 weeks (Litwin and Bridge, 1971). This discrepancy in APD values reported may be explained by differences in pacing protocols and restitution characteristics. Although both studies observed APD shortening when increasing pacing frequency (Litwin and Bridge, 1971; Lee et al., 2013), the pacing frequency for baseline APD measurements was higher in Lee et al. (2013) (3 Hz) than in Litwin and Bridge (1971) (0.5 Hz). This might explain the shorter baseline APD reported by the former compared to the latter. Walker et al. (2007) also applied higher pacing rates (3–6 Hz) than Litwin and Bridge (1971). However, although abnormal APs were reported in the BZ, the specific characteristics of these APs were not described.

The fact that APD shortening was found in feline 2 months after MI, but not in canine murine, and humans might be explained by differences in the specific coronary occlusion techniques implemented. For instance, the ligation technique employed in the feline experiments (Myerburg et al., 1982; Wong et al., 1982; Kimura et al., 1986, 1988; Pinto et al., 1997) was specifically developed to induce long term EP changes after MI (Myerburg et al., 1977), whereas the ligation technique employed in the murine experiments (Mills et al., 2005; Rutherford, 2013; Weigand et al., 2016) was designed to reduce infarct size and mortality rate after acute ischemia (Maclean et al., 1978), thus, likely attenuating EP remodeling in healed infarcts. Moreover, coronary occlusion followed by reperfusion was performed in canine (Spear et al., 1983) and the total repolarization time (APD_100_) was normal 5 and 14 days after MI, suggesting that this technique might also attenuate EP remodeling in healed infarcts. Is it worth noting that, reperfusion following coronary occlusion is typical in patients which are treated in hospital for MI. Although this technique may cause what is known as reperfusion injury, the canine results with reperfusion (Spear et al., 1983) point to a faster healing process resulting in less or even no EP remodeling in the clinical setting. This hypotheses is supported by the APD measured at the endocardial-lateral BZ in one patient with healed infarct, which was not significantly different than the APD of remote tissue (Dangman et al., 1982) and by the mean activation recovery interval values, as an APD surrogate, measured at the BZ of swine 5 weeks after MI, which show that values are not significantly different than in normal tissue, neither in the epicardium, nor in the endocardium (Denisko et al., 2017).

##### 2.2.1.2. Endocardial-central border zone

A thin rim (less than 800 μm Bakker et al., 1988) of myocardium is known to survive just below the endocardial surface overlying the core of the infarct scar in humans (Spear et al., 1979; Dangman et al., 1982; Bolick et al., 1986; Bakker et al., 1988; Smith et al., 1991), feline (Myerburg et al., 1982; Wong et al., 1982; Kimura et al., 1986, 1988) and rabbit (Walker et al., 2007), which we refer to as the endocardial-central BZ. Most APD measurements in this region show a longer APD compared with remote (normal) tissue (Spear et al., 1979; Dangman et al., 1982; Myerburg et al., 1982; Wong et al., 1982; Kimura et al., 1986). However, a later study by Kimura et al. (1988) show that APDs in the endocardial-central BZ are not significantly longer than the APD in remote tissue. The authors argue that this discrepancy is likely due to differences in experimental conditions. Specifically, their LV preparation was perfused through the coronary arteries (Kimura et al., 1988), whereas the preparations in the earlier studies were superfused (Myerburg et al., 1982; Wong et al., 1982; Kimura et al., 1986). When LV preparations are superfused, the cells in the mid-myocardium may be hypoxic, resulting in APD shortening. This effect was demonstrated in a recent study using computational models (Campos et al., 2012). Since the endocardial-central BZ is isolated from the mid-myocardium, as it overlies the core of dense fibrosis, this region is less affected by a hypoxic mid-myocardium. Consequently, the APD in the remote tissue is likely to be shorter than in the endocardial-central BZ, as reported in feline (Myerburg et al., 1982; Wong et al., 1982; Kimura et al., 1986) and human (Spear et al., 1979; Dangman et al., 1982) superfused preparations. Thus, considering the likelihood of such a significant experimental artifact highlighted by Kimura et al. (1988) and the fact that no ionic remodeling was found in the feline endocardial-central BZ (Kimura et al., 1986), we conclude that the APD in the endocardial-central BZ of healed infarcts is likely not significantly different from the normal myocardium.

Such experimental artifacts may also have affected the APD measurements reported in the feline endocardial-lateral BZ, which was shorter than both remote and endocardial-central BZ tissue (Myerburg et al., 1982; Wong et al., 1982; Kimura et al., 1986). However, APD values of the endocardial-lateral BZ were not reported in the coronary perfused feline preparations (Kimura et al., 1988). On the other hand, APD values in this region were normal in human superfused preparations (Dangman et al., 1982). Therefore, further experimental studies would be required to determine the effect of superfusion on the APD of the endocardial-lateral BZ.

#### 2.2.2. Structural remodeling

The presence of fibrosis is frequently reported in the BZ of healed infarcts (Myerburg et al., 1977; Spear et al., 1979; Gardner et al., 1985; Ursell et al., 1985; Bakker et al., 1988; Luke and Saffitz, 1991; Smith et al., 1991; Pogwizd et al., 1992; Kostin et al., 2003). While interstitial fibrosis would impair conduction transverse to fibers (Bakker et al., 1988; Pogwizd et al., 1992), thus, increasing anisotropy, the presence of patchy fibrosis locally blocks propagation both longitudinally and transverse to fibers, leading to non-uniform anisotropy throughout the tissue. Both types of fibrosis are associated with fractionated electrograms (Gardner et al., 1985; Ursell et al., 1985; Bakker et al., 1988; Pogwizd et al., 1992). In addition, fibers in the BZ of healed infarcts are often reported to be in disarray (Gardner et al., 1985; Ursell et al., 1985; Pogwizd et al., 1992; McGuire et al., 1996; Kostin et al., 2003; Pop et al., 2012). When increased and/or non-uniform anisotropy are combined with fiber disarray, conduction may appear isotropic and slow at the macroscopic scale, whereas local conduction slowing in the transverse direction may occur at the microscopic scale. In fact, slow and isotropic propagation was observed at the BZ of healed canine infarcts (Gardner et al., 1985; Ursell et al., 1985). On the other hand, although GJ lateralization was observed in patients with healed infarcts (Smith et al., 1991; Kostin et al., 2003), its role in propagation at the BZ remains to be fully determined, as previously discussed.

#### 2.2.3. Summary

The experimental findings listed in Table 1 clearly demonstrate that (1) the EP and structural properties of the BZ vary significantly from 3 days to 12 months after infarct, allowing a clear separation between the healing (3 days to 5 weeks) and healed phases (more than 5 weeks), (2) APD at the BZ is shorter than normal or remote tissue during the healing phase, (3) APD at the BZ in healed infarcts is not significantly different than normal or remote tissue (4) conduction slowing is mostly associated with the presence of fibrosis, which starts to appear at the BZ 7 days after infarct, (5) the BZ of healed infarcts is mostly marked by the presence of fibrosis and fiber disarray leading to slow conduction and loss of anisotropy, respectively.

## 3. Computational representations of the infarct border zone

Increased dispersion of repolarization has been associated with life-threatening arrhythmias (Kléber and Rudy, 2004; Clayton and Holden, 2005; Coronel et al., 2009). Repolarization dispersion is mainly determined by the spatial distribution of repolarization times in cardiac tissue, which are in turn determined by local APD and CV. Thus, changes in the spatial distribution of APD and CV may introduce repolarization heterogeneity, increasing dispersion of repolarization, in turn affecting predicted arrhythmia vulnerability. Experimental data presented in Table 1 shows that APD may be shorter or longer at the BZ depending on the stage of infarct healing, species and the specific BZ region, while slow conduction and fiber disarray were a common find at the BZ of healed infarcts. However, the consequences of changes in APD, CV and fiber orientation at the BZ on the spatial distribution and resulting dispersion of repolarization in the vicinity of the scar are unclear. Thus, we created idealized 2D computational models of scar and BZ, where APD, tissue conductivities and fiber orientation are modified at the BZ. We simulated steady-state activation and repolarization sequences and computed local activation and repolarization times and gradients of repolarization times. The cellular action potential and 2D tissue models are described in the next section followed by simulation results.

### 3.1. Idealized computational models

**Figure 2:**
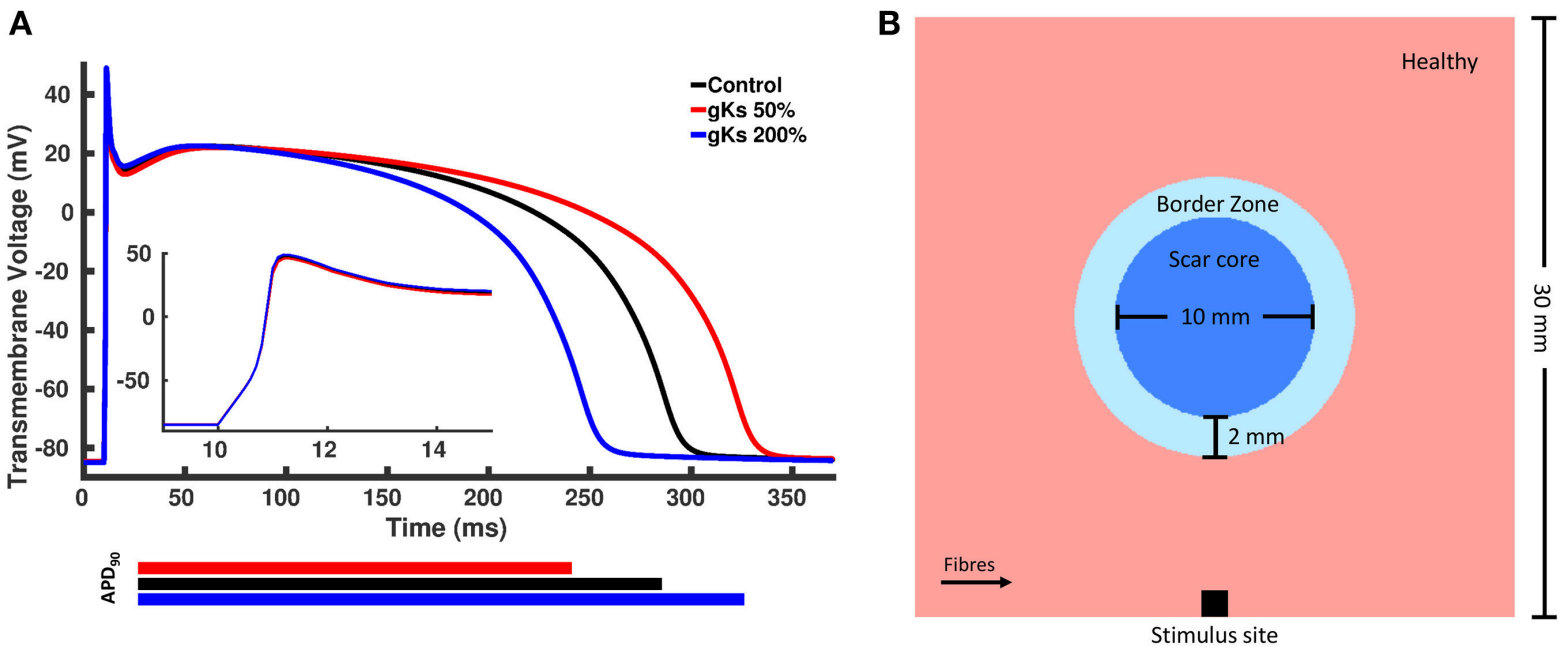
**(A)** Action potentials of the control and two modified models. The blue line represents the model with shorter action potential duration (APD) obtained by increasing the conductance of the slow rectifying potassium current, g_*Ks*_, to 200% of the control value, whereas the red line represents the model with longer APD obtained by decreasing g_*Ks*_ to 50% of the control value and the black line represented the control model with unchanged parameters. **(B)** Rectangular tissue model with a circumferential scar. The whole tissue measures 30 mm × 30 mm, where the scar core measures 10 mm in diameter and is surrounded by a 2 mm thick BZ. Healthy myocardium is represented in pink, scar core in dark blue and BZ in light blue. Fibers in healthy myocardium are always aligned with the x axis, whereas at the BZ, fibers are either aligned with the x axis or randomly oriented. Stimuli are delivered at the center of the bottom tissue edge.

We created modified AP models based on the Ten Tusscher model of human ventricular cells (ten Tusscher et al., 2006), where one model has a longer APD and the other has a shorter APD. A longer APD was obtained by decreasing the conductance of the I_*Ks*_ current, g_*Ks*_, to 50% of the control value, whereas a shorter APD was obtained by increasing g_*Ks*_ to 200% of the control value. We chose g_*Ks*_, as it yields the most significant effect on the APD of the Ten Tusscher model (Mirams et al., 2014). The APD of the shorter and longer APD models were approximately 40 ms shorter and longer than the control value, respectively, based on APD values found in the literature (Litwin and Bridge, 1971; Ursell et al., 1985). The APs generated by the two modified models as well as the control model with unmodified parameters are shown in Figure 2A.

A rectangular tissue setup with a circular scar was created to investigate changes in the spatial distribution of repolarization gradients for different computational representations of the BZ. Specifically, a 30 × 30 mm^2^ finite element (FE) grid of triangular elements with mean edge length of 50 μm was created. A schematic representation of our tissue setup is shown in Figure 2B. The scar core was modeled as an insulator by removing the elements defined as scar core from the FE grid. This simple representation was chosen over a more realistic anatomical representation of the myocardium and scar to avoid confounding factors, such as ventricular wall and scar shape, scar location and transmurality, as well as apico-basal and transmural fiber rotation.

The cardiac monodomain model was used to simulate electrical propagation in the tissue. Normal anisotropic bulk conductivities were set to 0.1890 and 0.0690 S/m in the longitudinal and transverse directions, respectively. Using an automatic parameterization approach (Costa et al., 2013), these conductivities yield velocities of 0.6 and 0.4 m/s, consistent with velocities in normal ventricular myocardium (Caldwell et al., 2009). The computational representations of the EP properties of the BZ were based on the findings listed in Table 1. These include: shorter, normal, or longer APD, normal conductivities, decreased transverse conductivity representing slow transverse propagation due to the presence of interstitial fibrosis, or decreased isotropic conductivities representing slow isotropic propagation, and horizontally or randomly oriented fibers representing normal fiber alignment and fiber disarray, respectively. The decreased transverse conductivity value was set to 10% of the normal value, as done in recent simulations studies (Arevalo et al., 2013, 2016), yielding a velocity of 0.12 m/s. The isotropic conductivities were computed based on the CV measured at the BZ with decreased transverse conductivities and randomly aligned fibers (0.4 m/s) using an automatic approach (Costa et al., 2013). The conductivity values used are shown in Table 2. A cell capacitance of 1μ*F*/*cm*^2^ and a surface-to-volume ratio of 0.14 μ*m*^−1^ were used in all simulations.

**Table 2:** Conductivity types and values used in the 2D simulations.

**Type**	**Longitudinal (S/m)**	**Transverse (S/m)**
Normal anisotropic	0.1890	0.0690
Decreased transverse	0.1890	0.0069
Decreased isotropic	0.0689	0.0689

The monodomain equation coupled with the Ten Tusscher ionic model was solved using the Cardiac Arrhythmia Research Package (CARP) (Vigmond et al., 2003, 2008). The cell model was stimulated 100 times prior to tissue simulations to achieve steady-state. The final state of gating variables was saved and given as input to the tissue simulations. The tissue was then stimulated 5 times at a constant cycle length of 500 ms to achieve steady-state. Activation time was computed as the time tissue reached a threshold of –20 mV and repolarization time was computed as the time tissue reached a threshold of –70 mV after depolarization. The repolarization gradient was computed as the magnitude of the spatial gradient of the repolarization time at each grid point.

### 3.2. Activation times

**Figure 3:**
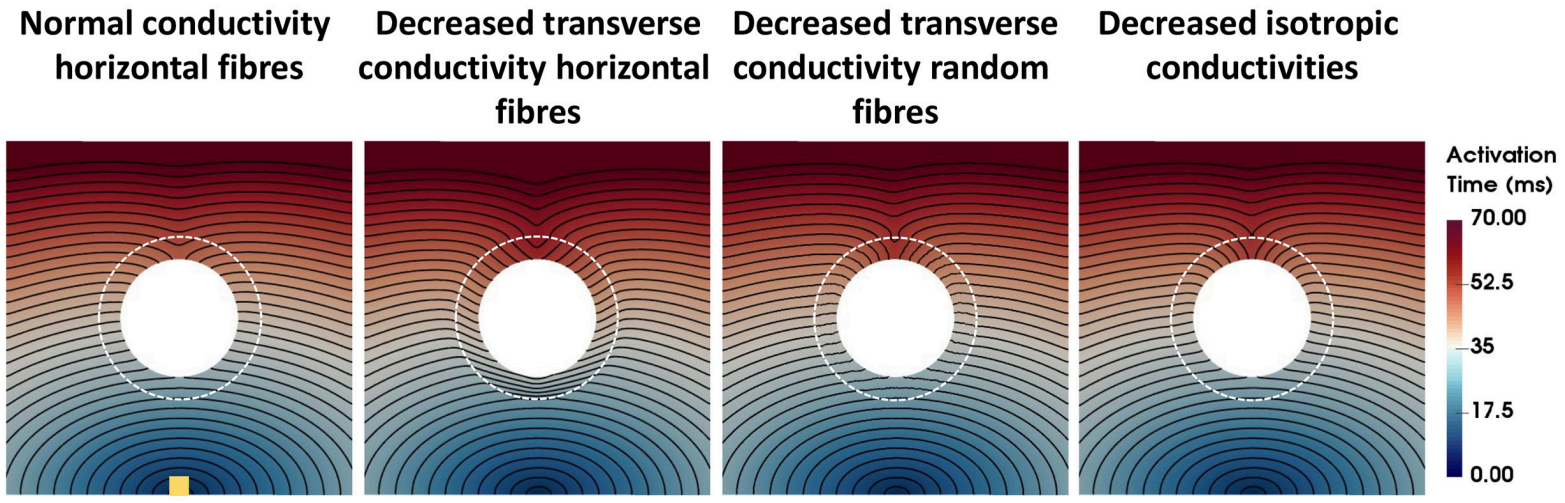
Activation times for four computational representations of the BZ with normal APD. The Scar core is shown as a white filled circle, whereas the interface of BZ with normal tissue is marked as a dashed white circle. The stimulus site is represented by the yellow square at the bottom left panel. The stimulus site was the same for all simulations. Activation times ranging from 0 ms (blue) to 70 ms (red) are shown for the four models. The columns show, from left to right, the models with normal conductivity, decreased transverse conductivity with horizontal fibers, decreased transverse conductivity with random fibers, and decreased isotropic conductivities in the BZ.

The activation times for four computational representations of the BZ with normal APD, the conductivities shown in Table 2, and horizontal and random fibers are shown in Figure 3. The activation sequences for the BZ with normal conductivities, with decreased transverse conductivities and random fibers, and with reduced isotropic conductivities are very similar to each other. Particularly, the activation sequences of the latter two are nearly identical. On the other hand, an abrupt transition from normal to very slow conduction (30% slower) when the BZ is represented with decreased transverse conductivities and horizontal fibers causes a significant activation delay. This effect is seen as crowding of isolines at the bottom of the BZ on the second left panel of Figure 3.

### 3.3. Repolarization times and gradients

**Figure 4:**
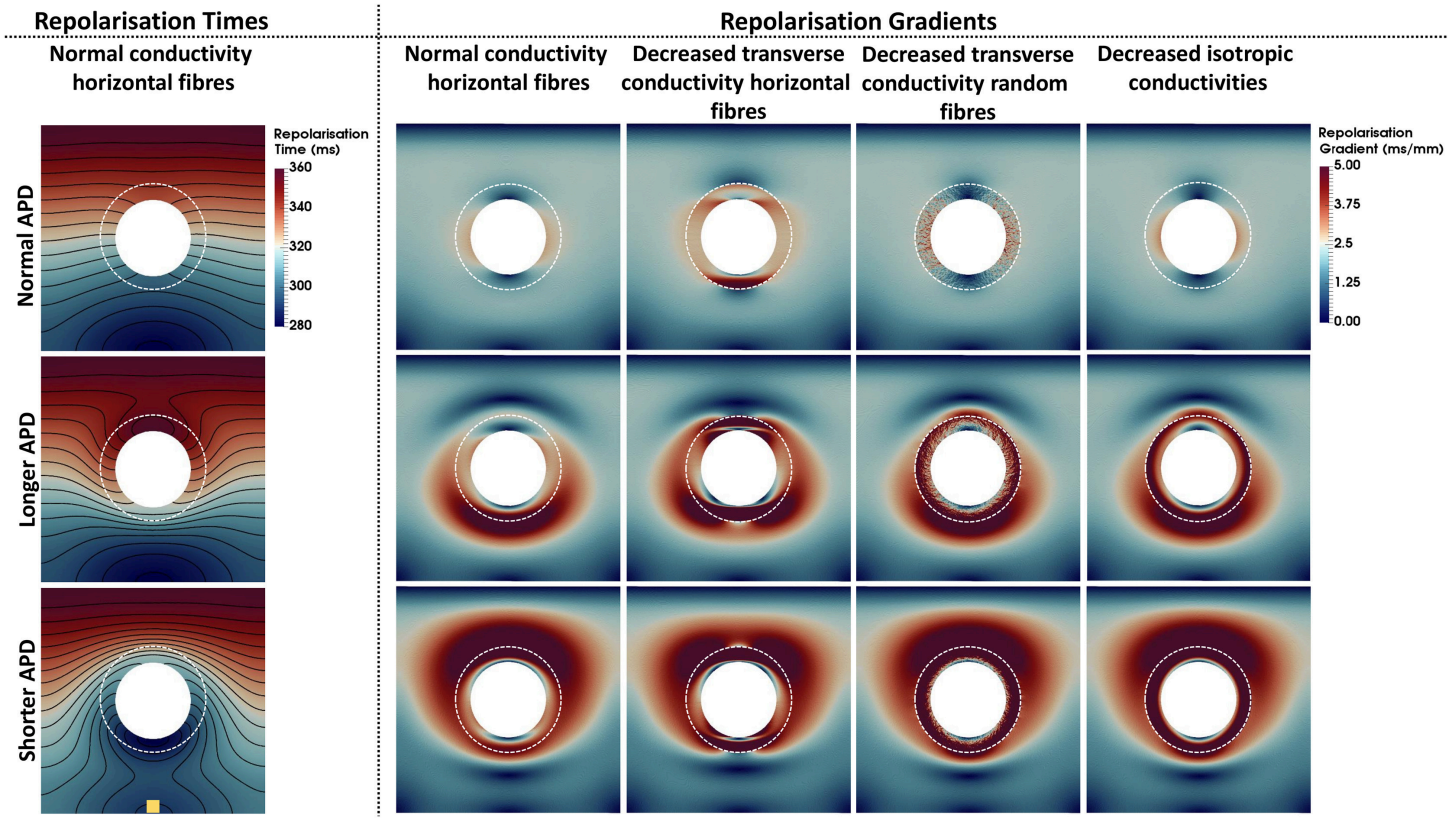
Repolarization times and gradients computed for the 12 computational representations of the BZ. The Scar core is shown as a white filled circle, whereas the interface of BZ with normal tissue is marked as a dashed white circle. The stimulus site is represented by the yellow square at the bottom left panel. The stimulus site was the same for all simulations. The left column shows the repolarization time maps. Repolarization times ranging from 280 ms (blue) to 360 ms (red) are shown for the models with normal conductivities and horizontal fibers. The second to fifth columns from left to right show the repolarization gradients. Gradients range from 0 ms/mm (dark blue) to 5 ms/mm (dark red). The upper, central, and bottom rows show the models where the APD was set to normal, longer, or shorter than normal tissue, respectively. The second to fifth columns show, from left to right, the models with normal conductivity, decreased transverse conductivity with horizontal fibers, decreased transverse conductivity with random fibers, and decreased isotropic conductivities in the BZ.

Repolarization times and gradients computed for each model are shown in Figure 4. The repolarization time maps shown in the left column describe the repolarization sequence for each AP model. Note that the presence of a longer or shorter APD at the BZ distorts the isolines, which accumulate at the bottom and top edges of the BZ for the longer and shorter APD models, respectively. Where the isolines accumulate, large repolarization gradients are seen in the second to fifth columns, left to right. Particularly, gradients above 5 ms/mm extend up to 50 mm around the BZ when APD is shortened at the BZ and up to 30 mm when APD is prolonged for all conductivities. Similar to the activation times, the different conductivities also have a less prominent effect on the spatial distribution of repolarization gradients. However, when the transverse conductivity is severely reduced thus reducing electrotonic load in the transverse direction, gradients become sharper and the spatial pattern is distorted. Particularly, decreased transverse conductivity introduces large sharp gradients at the top and bottom edges of the BZ when combined with horizontally aligned fibers, whereas, small patches of large gradients appear when decreased transverse conductivity is combined with randomly oriented fibers. Macroscopically, the spatial distribution of gradients of the model with decreased transverse conductivity and randomly oriented fibers is similar to the models with normal conductivity and decreased isotropic conductivities and horizontally aligned fibers. It is worth mentioning that the scale of gradients (0–5 ms/mm) was chosen for visualization purposes only. The minimum repolarization gradient required for unidirectional block was determined experimentally as 3.2 ms/mm (Laurita and Rosenbaum, 2000). The repolarization gradients in our simulations were often much larger than 3.2 ms/mm, being as large as 25 ms/mm at the edge of the BZ in the models with altered APD, decreased transverse conductivity and horizontal fibers.

## 4. Discussion

In this study, we presented a literature review on experimental data of the EP and structural properties of the infarct BZ. Experimental data show that BZ properties change over time after coronary occlusion. During infarct healing (<5 weeks), the majority of studies find that the BZ exhibits a shorter APD, slower MUV and decreased APA than normal and or remote tissue. Two weeks following occlusion fibrosis starts to appear and this coincides with conduction slowing. As the infarct heals (>5 weeks), APD, MUV and APA return to normal values in the majority of studies independent of species. We used idealized computational models to study the impact of these different electrophysiological and structural changes reported to occur within the BZ on repolarization gradients within the vicinity of regions of infarct scar. Our simulation results show that repolarization gradients, as a proxy for arrhythmogenic risk, are highly dependent on the specific conductivity and APD values as well as fiber orientation used to represent the BZ. Unsurprisingly, those models with specific APD alterations assigned to BZ showed the largest repolarization gradients. These findings thus highlight the need to accurately model the BZ in computer models that are based on experimental data.

### 4.1. Impact of different computational representations of the infarct border zone on activation and repolarization

The experimental findings listed in Table 1 demonstrate that APD is shorter than normal during infarct healing, whereas APD in healed infarcts is likely normal. When implementing local differences in APD in our computer models, repolarization gradients as large as 25 ms/mm appear in and around the BZ. In general, the intrinsic APD of a cell, i.e., the APD defined by ionic properties, is smoothed out when cells are coupled due to electrotonic interaction between neighboring cells. This so-called electrotonic effect is modulated by tissue conductivity, with larger conductivities leading to more smoothing. In fact, despite the large difference in APD between cells of healthy and BZ tissue (~40 ms), repolarization gradients due to longer or shorter APD are smoothed out between the two regions for all BZ conductivites, but to different degrees (Figure 4). Particularly, reduced transverse conductivity leads to decreased electrotonic load in the transverse direction, introducing sharp gradients at the BZ and increasing spatial heterogeneity. This effect is observed both with horizontally and randomly aligned fibers, however, large gradients are restricted to small localized regions within the BZ when fibers are randomly aligned. On the other hand, APD differences are smoothed out to a larger extent with normal and reduced isotropic conductivities.

It is worth mentioning that, the sharp gradients observed in the case of decreased transverse conductivity are a result of an extreme reduction in conductivity. To investigate whether a gradual transition between healthy and BZ tissue would affect results, we performed one additional simulation, where we implement a smooth transition between healthy and BZ tissue for the case of decreased transverse conductivity (see Figure S1 in Supplementary Material). This approach resulted in a slightly smoother repolarization gradient, but did not qualitatively alter our results and conclusions. Moreover, since the exact nature of such a gradual reduction in conductivity is not currently known, employing such approach would require a detailed modeling investigation which is out of the scope of this study.

Several experimental studies listed in Table 1 report decreased CV at the BZ ranging from 50% (Mills et al., 2005) to 10% (Gardner et al., 1985; Ursell et al., 1985) slower. However, decreased conductivities yielding slower conduction in the BZ only significantly affected activation times (Figure 3) and repolarization gradients (Figure 4) in our simulations when the transverse velocity was reduced to 30% of the normal value (0.12 m/s instead of 0.4 m/s).

In general, slow conduction in the BZ is associated with the presence of interstitial and patchy fibrosis as well as fiber disarray during late infarct healing and in healed infarcts. Our simulations show that the combination of interstitial fibrosis (represented by decreased transverse conductivity) and fiber disarray (represented by randomly aligned fibers) lead to spatial distributions of repolarization gradients that are macroscopically very similar to those obtained with decreased isotropic conductivities. These findings thus indicate that conduction in the BZ of infarcts older than 2 weeks is likely slow and isotropic, as reported in the canine epicardial BZ of healed infarcts (Ursell et al., 1985).

Based on experimental findings and our simulation results, we conclude that the macroscopic EP properties of BZ are best represented by (1) reduced MUV, APA and APD without conductivity changes during early (<7 days) infarct healing, (2) shorter APD and decreased isotropic conductivities during late infarct healing (7 days to 5 weeks), and (3) normal APD and decreased isotropic conductivities in healed (more than 5 weeks) infarcts, regardless of the specific sub-region (endo-central, endo-lateral, epi).

### 4.2. The role of myofibroblasts

Myofibroblasts are largely responsible for tissue repair following cardiac injury, particularly infarction (Baum et al., 2011). Recently, myofibroblasts have been found to form hetero-cellular connections at the BZ of healed cryoinjury scars (Quinn et al., 2016), however, their contribution to the electrical properties of the infarct BZ remains unclear. Due to their more positive resting potential relative to cardiomyocytes, myofibroblasts coupled to cardiomyocytes may act as an electrical sink affecting excitability, CV and APD (Rohr, 2012). These changes might be associated with increased likelihood of arrhythmia (Rohr, 2012) depending on myofibroblast density (McDowell et al., 2011).

Although the data presented in Table 1 did not explicitly include myofibroblasts, any effect they might have had in the electrical properties of the BZ would be implicitly accounted for in measurements of AP characteristics and CV. Thus, we do not expect the presence of myofibroblasts to significantly alter our conclusions based on the experimental data and simulation results presented in this study.

### 4.3. Comparison with recent simulation studies

Recent simulation studies using image-based models of infarct have relied on experimental data to model the EP properties of the BZ. Specifically, the BZ was modeled with reduced MUV and APA (Arevalo et al., 2013, 2008, 2016; McDowell et al., 2011; Rantner et al., 2012; Ringenberg et al., 2012; Ashikaga et al., 2013; Deng et al., 2015, 2016) and longer APD (Arevalo et al., 2013, 2008, 2016; McDowell et al., 2011; Ng et al., 2012; Rantner et al., 2012; Ringenberg et al., 2012; Ashikaga et al., 2013; Deng et al., 2015, 2016). Altered AP characteristics in the BZ were obtained by modifying individual ionic currents (Arevalo et al., 2013, 2008, 2016; McDowell et al., 2011; Rantner et al., 2012; Ringenberg et al., 2012; Ashikaga et al., 2013; Deng et al., 2015, 2016) based on experimental measurements of canine 5 days after MI (Pu and Boyden, 1997; Jiang et al., 2000; Yao et al., 2003; Dun et al., 2004), which lead to a longer APD (Decker and Rudy, 2010), or based on data obtained from feline 2 months after MI (Myerburg et al., 1982). However, while decreased MUV and APA are reported in canine (Spear et al., 1979; Ursell et al., 1985) during infarct healing, most experimental data listed in Table 1 show a shorter APD in the same phase, particularly 5 days after infarct (Spear et al., 1983; Gardner et al., 1985; Ursell et al., 1985; Baba et al., 2005).

The transverse conductivity was reduced to 10% of the normal value (Arevalo et al., 2008, 2013, 2016; McDowell et al., 2011; Rantner et al., 2012; Ashikaga et al., 2013; Deng et al., 2015, 2016) to represent decreased gap junctional conductance in the canine epicardial BZ 5 days after infarct (Yao et al., 2003). Although the relationship between conduction slowing transverse to fibers and GJ remodeling is not entirely clear (Cabo et al., 2006), slow transverse conduction was reported in murine 7 days after MI (Mills et al., 2005), where the authors report the presence of fibrosis, but do not report fiber disarray. This suggests that representing the BZ with reduced transverse conductivity may be appropriate in the presence of fibrosis without fiber disarray. The BZ was also represented with conductivity 3 times slower than healthy myocardium (Ng et al., 2012), consistent with the values estimated in porcine ventricles (Pop et al., 2012). However, the authors used an isotropic setup, where changes in fiber orientation cannot be represented.

### 4.4. Consequences for arrhythmia simulations

Re-entrant arrhythmias in patients with MI typically originate at the scar, particularly at the BZ (Bakker et al., 1988; Pogwizd et al., 1992). The core of the scar alone creates an area of anatomical, or fixed, conduction block, providing a substrate for anatomical re-entry. In the case of anatomical re-entry, the minimal length of the re-entrant pathway has to exceed the wavelength of excitation (Rohr et al., 1998), which can be defined as the product of CV and effective refractory period, which is largely determined by APD. The experimental data presented in this study shows that the BZ is characterized by short APD during infarct healing and slow conduction both during healing and in healed infarcts. Thus, the specific EP properties of the BZ may shorten the wavelength and increase the likelihood of arrhythmia.

While slow conduction is in general associated with increased arrhythmogenesis (Rohr et al., 1998; Kléber and Rudy, 2004), reduction of the transverse conductivity leading to slow conduction in the direction transverse to fibers, will shorten the wavelength in one direction increasing the chance of unidirectional block and re-entry (Saffitz and Kléber, 2012). This scenario may cause a type of re-entry know as anisotropic re-entry, which is characterized by conduction block in the transverse direction, owing to reduced transverse conductivity, but successful conduction in the longitudinal direction (Spach and Josephson, 1994). Decreased transverse conductivities also lead to large repolarization gradients in the BZ, which may also be associated with functional re-entry (Kléber and Rudy, 2004), although to a lesser extent. On the other hand, altered APD at the BZ leads to much larger repolarization gradients. Particularly, a shorter APD will also shorten the wavelength, making the formation of a sustained re-entrant circuit more likely. On the other hand, a longer APD at the BZ may be arrhythmogenic in the presence of an isthmus (Connolly and Bishop, 2016), where propagation is initially blocked, due to increased refractoriness, but might enter the isthmus creating a re-entrant circuit.

Increased refractoriness may also be caused by reduced *I*_*Na*_. Specifically, reduced *I*_*Na*_ in canine 5 days after MI (Lue and Boyden, 1992; Pu and Boyden, 1997; Baba et al., 2005) decreases excitability leading to post-repolarization refractoriness and thus an elongated effective refractory period (ERP) (Pu and Boyden, 1997). Thus, while the APD of a BZ cell may be shorter at this stage of MI the ERP may be longer (Gough et al., 1985; Pu and Boyden, 1997) potentially affecting arrhythmia mechanisms. While we have not included changes in *I*_*Na*_ in our computational models, the role of post-repolarization refractoriness in propagation block has been investigated in recent simulation studies (Cabo and Boyden, 2003; Decker and Rudy, 2010). In fact, post-repolarization refractoriness due to reduced *I*_*Na*_ was shown to be a major determinant of the vulnerable window for conduction block under fast pacing rates (Decker and Rudy, 2010).

In the case of chronic infarcts, our simulations show that the presence of interstitial fibrosis and fiber disarray may lead to large but localized repolarization gradients, as these gradients are mostly smoothed out by electrotonic interaction with the healthy tissue. In certain scenarios, these localized gradients may be sufficient to cause uni-directional conduction block of a nearby ectopic focus, which are known to be more likely to occur in the BZ tissue (Bakker et al., 1988; Pogwizd et al., 1992). Alternatively, they may cause heterogeneous conduction slowing of a preceding wavefront or potentially initiate wavebreak. Overall slow conduction in the BZ leading to wavelength shortening may contribute to anatomical re-entry, as an ectopic focus originating near the scar (Bakker et al., 1988; Pogwizd et al., 1992) may encounter a pathway larger than the wavelength and, thus, generate a re-entrant circuit. Thus, in this scenario, the anatomical properties of the scar and its BZ may play a more prominent role in re-entry than AP characteristics.

In summary, the remodeled infarct BZ may be associated with increased risk of re-entrant arrhythmias and the specific mechanism of re-entry will depend on the EP and structural properties of the BZ. These, in turn, depend on the specific stage of infarct healing. Thus, accurately modeling the EP properties of the BZ according to the stage of MI being modeled is crucial for arrhythmia simulations which aim at understanding arrhythmia mechanism and, particularly, predicting arrhythmia risk in patients with infarct.

## Author contributions

Jointly developed the structure and arguments for the paper: CMC, MB, SN. Performed the literature review and experiments: CMC. Analyzed the data: CMC, MB, SN. Wrote the first draft of the manuscript: CMC. Contributed to the writing of the manuscript: MB, SN, GP, CR. Agree with manuscript results and conclusions: CMC, MB, SN, GP, CR. Made critical revisions and approved final version: CMC, MB, SN, GP, CR. All authors reviewed and approved of the final manuscript.

### Conflict of interest statement

The authors declare that the research was conducted in the absence of any commercial or financial relationships that could be construed as a potential conflict of interest.
