# Supplementary figures and images for: Modeling the Electrophysiological Properties of the Infarct Border Zone
Source: Front Physiol. 2018 Apr 9;9:356. doi: 10.3389/fphys.2018.00356 (PMC5900020; doi:10.3389/fphys.2018.00356)

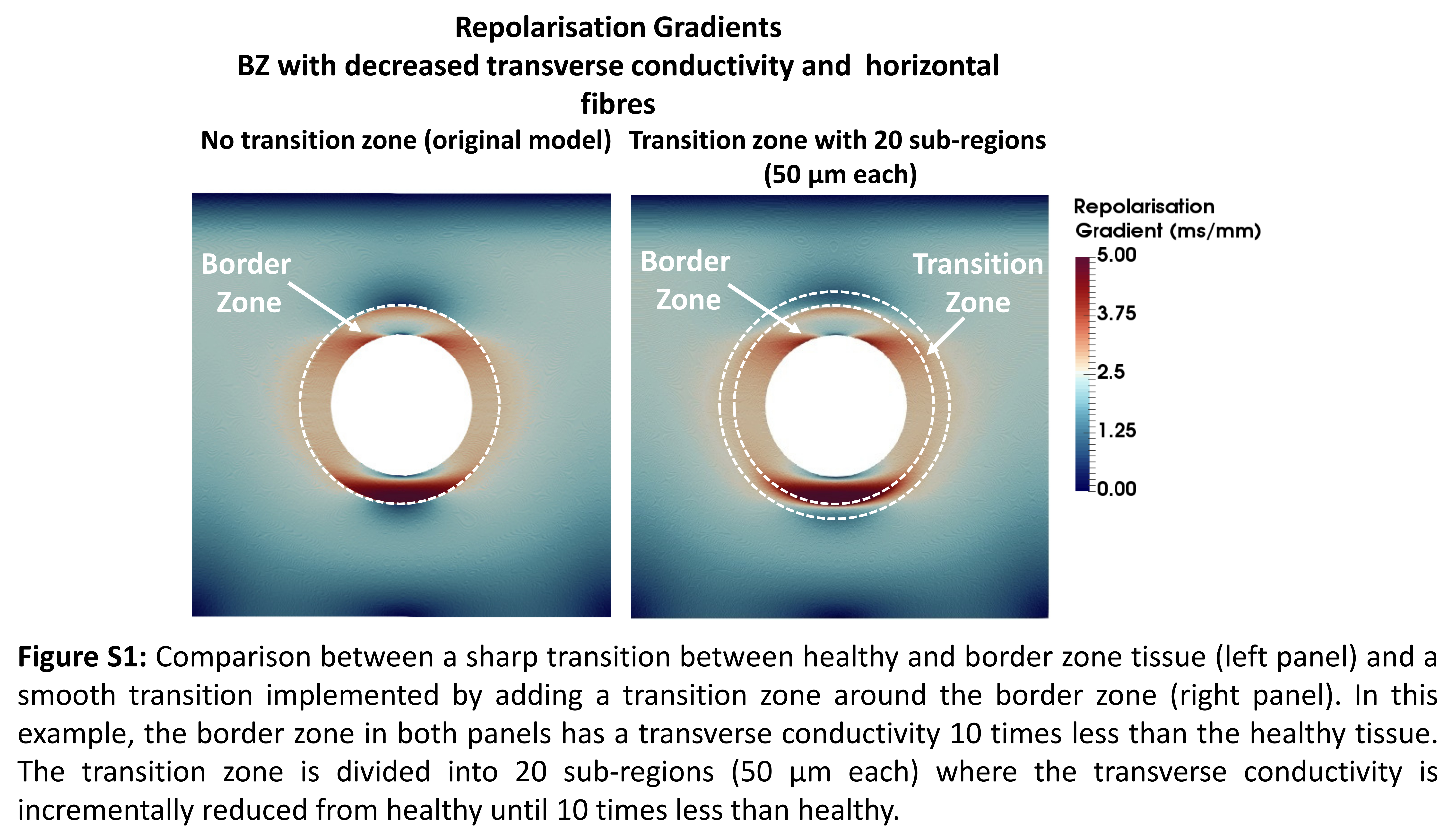

Supplement: Supplementary file 1 [file Image1.jpg]
